# Functional characterization of an inducible bidirectional promoter from *Fusarium oxysporum* f. sp. *cubense*

**DOI:** 10.1038/s41598-020-59159-0

**Published:** 2020-02-11

**Authors:** Ashutosh Dash, Vartika Gurdaswani, Jacinta S. D’Souza, Siddhesh B. Ghag

**Affiliations:** School of Biological Sciences, UM-DAE Centre for Excellence in Basic Sciences, Kalina campus, Santacruz (East), Mumbai 400098 India

**Keywords:** Expression systems, Transcriptional regulatory elements

## Abstract

Bidirectional promoters (BDPs) are regulatory DNA sequences (~1000 bp long) intervening two genes arranged on opposite strands with their 5′ ends in close proximity. These genes are mostly co-expressed; but, instances of anti-correlation and independent transcription have been observed. In fungal systems, BDPs have shown to provide an improved genetic circuit by assembling and regulating transcription of different genes of a common metabolic pathway. We have identified an intergenic region (1063 bp) from the genome of *Fusarium oxysporum* f. sp. *cubense* (*Foc*), a banana root pathogen. This intergenic region regulates the expression of a gene pair required for the breakdown of hemicellulose. For characterization, it was cloned into pCSN44 vector backbone between two reporter genes, namely β-glucuronidase (GUS) and enhanced green fluorescent protein (EGFP). The newly formed vector was transformed into *Foc* and tested for its bidirectional expression activity. Using histochemical staining and fluorescence microscopy, the kinetics for both, GUS and EGFP expression were tested under different growth conditions respectively. The activity was differentially regulated by inducers such as xylan, arabinogalactan and pectin. This is the first report on the isolation of the intergenic region with inducible bidirectional promoter activity from *Fusarium*. Characterization of such BDPs will find applications in genetic engineering, metabolic engineering and synthetic biology using fungal systems.

## Introduction

A bidirectional promoter is an intergenic region of ~1–1.5 kb that regulates the flanking protein coding gene pair^1,2^. Known for many years, BDPs have been identified in all kingdoms^3–8^. Recent advancement in sequencing and genome annotation technologies have aided in understanding the organization and importance of BDPs. In human genome, 10% of the genes represent divergently transcribed gene pairs separated by an intergenic DNA fragment less than 1000 base pairs^4^. There has been a strong interest in identifying suitable regulatory elements expressing heterologous or native proteins in host systems among which unidirectional promoters attracted more attention. Unidirectional promoters which are constitutive, inducible and tissue-specific have been isolated and used for genetic improvement and recombinant protein expression. Albeit, using BDPs for metabolic engineering and synthetic biology is an advantage wherein the cumulative expression of the two genes can be quantitatively regulated^9^. Moreover, the negative impact of using multiple unidirectional promoters in gene pyramiding is also abridged^10^. Bidirectional promoter activity that generate cryptic unstable transcripts regulating transcription has been identified in yeast^5,6^. Vogl and co-workers^11^ generated a collection of 168 synthetic BDPs in the methylotrophic yeast *Pischia pastoris* using its natural histone promoters as an engineering template. BDPs are abundant in plants, and divergent gene pairs were identified in most plant species ranging from *Arabidopsis* to rice and *Populus* wherein these account ~30% of their genomes^12^. The *Arabidopsis* genome harbors a large proportion (13.3%) of bidirectional gene pairs with an intergenic region of about 1–1.5 kb^13^. A total of 2106, 1242, and 613 BDPs were confirmed in rice, *Arabidopsis* and *Populus*, respectively. Most of the bidirectional gene pairs identified are functionally associated with their intergenic regions enriched of regulatory elements that respond to a particular inducer. These inducible BDPs could be tissue-specific thereby rendering appropriate for expressing proteins in the host at a particular stage of their growth cycle. This will therefore prevent any developmental anomalies associated with expressing large amounts of heterologous proteins. A 1258 bp intergenic region between *At4g35985* and *At4g35987* genes of *Arabidopsis* contains a number of environmental stress-responsive and tissue-specific *cis*-regulatory elements which showed differential regulation of two genes under study^14^. A 1429 bp intergenic region between the genes *Ghrack1* and *Ghuhrf1* in *Gossypium hirsutum* was cloned and used for expressing reporter genes in *Arabidopsis*^15^. A BDP was built by arranging in tandem three copies of a modified enhancer from the promoter region of the *Arabidopsis PLDZ2* gene, which strongly responds to phosphate starvation. The induction of transcription is controlled by the availability of phosphate in the medium^16^. Such regulatory sequences work as a circuit switch to drive the expression or stop transcription.

Filamentous fungi are one of the best eukaryotic systems known to express recombinant proteins, especially enzymes of industrial scale in the cheapest possible means^17^. Moreover, filamentous fungi are genetically modified to restructure the metabolic fluxes which eventually results in expression and secretion of the protein of interest into the medium, making downstream processing easier. Bidirectional promoters would be of great advantage in this aspect. Few BDPs have been identified and isolated from fungi. Most of these promoters are seen to co-regulate functionally related genes such as those involved in a common metabolic pathway. Most of the secondary metabolite genes are clustered in the fungal genome and few of them are regulated by divergent promoters. The *Aspergillus nidulans* penicillin biosynthesis genes *acvA* and *ipnA* are divergently oriented and separated by an intergenic region of 872 bp^18^. The *pcbAB* and *pcbC* genes involved in penicillin biosynthesis in *Penicillium chrysogenum* and *Penicillium nalgiovense* are expressed from a 1.16 kb BDP region which is controlled by both nutritional and developmental factors^19^. The nitrite reductase (*niiA*) and nitrate reductase (*niaD*) genes in *Aspergillus nidulans* are transcriptionally regulated by a 1.2 kb intergenic region^20^. This intergenic region behaves as a switch; ‘on’ state in presence of nitrate and ‘off’ in presence of ammonium and glutamine. This nitrate-inducible promoter was used to express the transcription factor AflR and its cofactor AflS that were responsible for activating eight sterigmatocystin (precursor for aflatoxin B1) promoters for sterigmatocystin production in *Aspergillus nidulans*^21^. Further, this genetic circuit was used to produce β-carotene in *Aspergillus nidulans* from the carotenoid gene cluster of *Fusarium fujikuroi*^21^. Moreover, these BDPs are regulated by activators, transcription factors and enhancers that facilitate binding of the protein complexes driving expression of the gene pair. Modification of histones, positioning of the nucleosomes and binding of the transcription factors regulating BDP activity have been studied in model fungal systems such as *Aspergillus nidulans* and *Saccharomyces cerevisiae*^22–25^.

*Fusarium oxysporum* species complex are common soil pathogens that infect most crop plants causing wilt disease. It enters through the root system and eventually reaches the vascular tissues^26^. During infection, it releases an array of extracellular enzymes that degrade the plant cell wall for its progression. These enzymes are triggered in the presence of the inducer compound most probably derived from the host plant. Xylan is an important component of plant cell wall and the virulent organisms possess enzymes to break xylan as one of the virulence factors. In this study, we have identified an intergenic region shared by divergent genes encoding a putative beta-xylosidase and arabinoxylan arabinofuranohydrolase placed head to head. This intergenic region was able to drive the expression of reporter genes GUS and EGFP demonstrating bidirectional activity of this DNA fragment. Moreover, this region was inducible and displayed differential transcription of the two reporter genes. This is the first study that reports the isolation of a BDP from an economically important phytopathogen *Fusarium oxysporum*.

## Methods

### Primers used in this study

The primers used in this study are listed in Table 1.

### Culture conditions

The strain used in this study, *Fusarium oxysporum* f. sp. *cubense* (*Foc*) race 1 was kindly provided by Dr. T. R. Ganapathi from Bhabha Atomic Research Centre, Mumbai and grown in potato dextrose medium. For condial preparation, *Foc* was grown in potato dextrose agar (PDA) medium incubated at 30 °C for 8–10 days whereas for liquid cultures potato dextrose broth (PDB) was used as medium and cultures shaken at 160–200 rpm at 30 °C.

### Isolation and *in silico* analysis of the bidirectional promoter from *Foc*

The sequence from *Foc* race 1 contig1365 whole genome shotgun sequencing database with GenBank accession no AMGP01001365 was identified wherein this intergenic sequence was located between two genes involved in xylan metabolism. This intergenic region was isolated from *Foc* genomic DNA using primer set PxyFw/PxyRv (Table 1) and sequenced. The sequence was analyzed for promoter elements using online software such as PLANT Care, PlantPAN 2.0 and PLACE.

**Table 1:** Primers used in this study.

Primer name	Primer sequence
Pxy Fw	5′ CCAGCGGCCGCTTTGGAGCCGTGAAGAACTAAAG 3′
Pxy Rv	5′ TTC GCTAGCCTTGAAGATGTCTCTCGAAATAGC 3′
EGFP Fw	5′ TTCGCTAGCATGGTGAGCAAGGGCGAGG 3′
EGFP Rv	5′ CAACCCGGGCTGTCTGGTCTTCTACACG 3′
GUS Fw	5′ TTCAGATCTTTATTGTTTGCCTCCCTGCTG 3′
GUS Rv	5′ TTAGCGGCCGCATGTTACGTCCTGTAGAAACC 3′
GAter Fw	5′ TTCGAGCTCCTGTCTGGTCTTCTACACGAA 3′
GAter Rv	5′ CCAAGATCTACAATCAATCCATTTCGCGATAG 3′

### Construction of the vector for *Foc* transformation

The intergenic region (Pxy) was amplified from the genomic DNA of *Foc* using primers PxyFw/PxyRv (Table 1) and digested using *Not*I and *Nhe*I sites. EGFP along with the glucoamylase gene terminator was amplified using primers EGFPFw/EGFPRv (Table 1) from pCSN44eGFP vector gifted by Prof. N. S. Punekar (IIT-B, Mumbai) and digested using *Nhe*I and *Xma*I. These two digested fragments were inserted into the vector backbone pCSN44 digested previously using *Not*I and *Xma*I in a three-way ligation reaction to give pCSN44Pxy-eGFP vector. The pCSN44Pxy-eGFP vector was digested using *Not*I and *Sac*I and used for further cloning. The β-glucuronidase gene was amplified from the vector pBI121 (AF485783.1) using the primers GUSFw/GUSRv (Table 1) and digested by *Not*I and *Bgl*II. The glucoamylase gene terminator was amplified from pCSN44eGFP using primers GAterFw/GAterRv (Table 1) and digested using *Bgl*II and *Sac*I restriction enzymes. These two fragments were cloned into the digested pCSN44Pxy-EGFP vector to form the pCSN44Pxy-EGFP-GUS vector. This newly constructed vector was purified and sequenced.

### *Foc* transformation and selection of the transformants

*Foc* spheroplasts were used for transformation^27^. The spheroplasts were prepared by first growing the *Foc* culture in PDA at 25 °C for 8–12 days. Conidia were harvested from this plate using Tween 80 solution (0.005% v/v) and separated from the mycelia by filtering it through the absorbent cotton bed. Approximately, 10^7^ spores/mL were inoculated into PDB and incubated at 30 °C and shaken at 200 rpm for 22–23 hours. The following day, mycelia were separated using four layers of cheese-cloth and washed several times with chilled distilled water followed by chilled osmotic medium (0.27 M CaCl_2_ and 0.6 M NaCl). The washed mycelia were transferred to a flask containing osmotic medium, lysing enzyme [900 mg/mL] (Novozyme, USA) and bovine serum albumin fraction V [60 mg/mL] (Sigma, USA) and mixed to form a homogenous slurry. This mixture was incubated in a water bath at 37 °C for 2–3 hours. The reaction mixture was swirled vigorously to release protoplast from the mycelial debris. This reaction mix was filtered through mira cloth (Calbiochem, USA) to separate the spheroplasts from the mycelial debris. The spheroplasts were pelleted by centrifuging at 1811 g in a swinging bucket rotor for 10 min. The pellet was washed with 1X STC solution (1.2 M D-sorbitol, 50 mM CaCl_2_ and 35 mM NaCl). The supernatant was discarded and the pellet was re-suspended in 140 µl of sterile 1X STC. Five micrograms of linearized plasmid DNA along with equal volume of 2X STC and 50 µl 50% w/v PEG (t) (2X STC: 50% w/v PEG 8000::1:1) was added to the spheroplasts and incubated on ice for 30 min. After incubation, 1 mL of PEG(t) was added to the mixture and incubated at room temperature for 30 min. This mixture was spread on to PDA medium with 27.38% sucrose and incubated at room temperature. After 16–18 h, the plates were overlaid with PDA medium supplemented with hygromycin-B (50 µg/mL) and incubated at 30 °C for 2–3 days till the appearance of transformants on the plates. The putative transformants were grown on PDA medium supplemented with hygromycin and screened by PCR using primers PxyFw/EGFPRv and GAterFw/GUSRv (Table 1). Since the growth seemed more comparable with the wild-type (WT) transformant T17 was chosen for further analysis.

### Histochemical β-glucuronidase (GUS) assay

The histochemical GUS assay was performed as described by Jefferson and co-workers^28^. *Foc* WT and T17 conidia were inoculated in PDB medium and shaken for 72 h at 160 rpm/30 °C. The mycelia were filtered and washed with sterile distilled water to remove remnants of the PDB medium. Equal amount of mycelia were inoculated into minimal medium supplemented with different carbon sources such as 1% xylan from beechwood (SRL, India), 1% arabinogalactan from larch wood (Sigma, USA), 1% pectin from citrus peel (Sigma, USA), 1.5% soluble starch (Sigma-Aldrich, USA), 1.35% D-xylose (Himedia, India) and 3% sucrose (Calbiochem, USA). The mycelial samples were collected after 3, 8, 12 and 24 h and incubated in GUS buffer overnight at 37 °C. Post-incubation the mycelial samples were clarified using 70% ethanol and observed for indigo coloration.

### 4-Methyl-umbelliferyl- β-D-glucuronide (MUG) assay

The WT and T17 *Foc* strains were grown in PDB medium for 72 h at 30 °C in an orbital shaker. The mycelia were separated using cheese cloth and washed thoroughly with sterile distilled water to remove any traces of the medium. The washed mycelia were inoculated in minimal medium supplemented with one of the carbon sources (xylan, arabinogalactan and pectin). The flasks were incubated for 12 h at 30 °C in an orbital shaker and used for MUG assay^28^. The mycelia were again separated, weighed and crushed using liquid nitrogen to fine powder. This powder was suspended in chilled 1X extraction buffer (10 mM EDTA, 10 mM β-mercaptoethanol, 0.1% sodium n-lauryl sarcosine, 0.1% Triton X-100 prepared in 50 mM sodium phosphate buffer, pH 7.0). The suspension was centrifuged at 18,407 *g* at 4 °C; supernatant was used for MUG assay and total protein estimated by Bradford method. For MUG assay, 150 µl of 4-Methylumbelliferyl-β-D- glucuronide hydrate (MUG) substrate [2 mM 4-MUG dissolved in 1X extraction buffer] (Sigma, USA) was diluted with 75 µl extraction buffer followed by equilibration at 37 °C. The reaction was started by adding 75 µl of the extract, incubated at 37 °C and terminated at an internal of 10 min up to 70 min using 200 mM sodium carbonate. The fluorescence intensity (FI) was measured after every time point using Cary Eclipse Fluorescence Spectrophotometer (Agilent, USA) with an excitation and emission wavelength of 365 nm and 455 nm respectively. The amount of 4-Methylumbelliferone [4-MU] (Sigma, USA) produced in this reaction was calculated with respect to FI vs. 4-MU concentration graph. 4-MU standard graph was obtained by measuring the FI of the solution containing 4-MU in 200 mM sodium carbonate solution from 0–100 nM. Each experiment was performed twice with three technical replicates per experiment.

### Fluorescence microscopy for enhanced green florescent protein (EGFP) detection

For EGFP detection, the WT and T17 *Foc* strains were grown in PDB and inoculated in minimal medium supplemented with different carbon sources as described in section 2.5. The mycelia were observed under oil immersion 100X objective Nikon Eclipse N*i* fluorescence Upright microscope (Nikon, Japan) 3, 8, 12 and 24 h post-inoculation. A 480/30 wavelength light obtained through an FITC filter set was used for EGFP excitation. The images were captured using the Nikon DS-Q*i*2 monochrome camera (Nikon, Japan).

### EGFP estimation using fluorimetry

The WT and T17 *Foc* strains were grown in PDB medium for 72 h, at 30 °C in orbital shaker and mycelia were separated using cheese cloth. This mycelia were washed with sterile distilled water and inoculated in minimal media containing different carbon sources such as xylan, arabinogalactan, pectin, xylose, starch and sucrose as described previously. After growing them for 12 h, at 30 °C in an orbital shaker, 2 ml of the growth medium containing the mycelia was used for measuring the EGFP fluorescence, with an excitation of 488 nm and emission of 509 nm using respective medium as blank. For the zero-hour WT and T17 time point samples, PDB medium was used as blank. The mycelial suspension after measuring fluorescence intensity was harvested at 21,130 *g* for 10 min and the wet weight was measured. The EGFP expression was calculated as the FI per mg of wet weight of mycelia. Each measurement was done in triplicates and the experiment was repeated twice.

### Statistics

Two-way ANNOVA Tukey’s multiple comparison test using GraphPad Prism 8.0.2. (*P < 0.05; **P < 0.01; ****P < 0.0001) was conducted for all experimental values.

## Results

### *In silico* analysis and isolation of the bidirectional promoter from *Foc*

**Figure 1:**
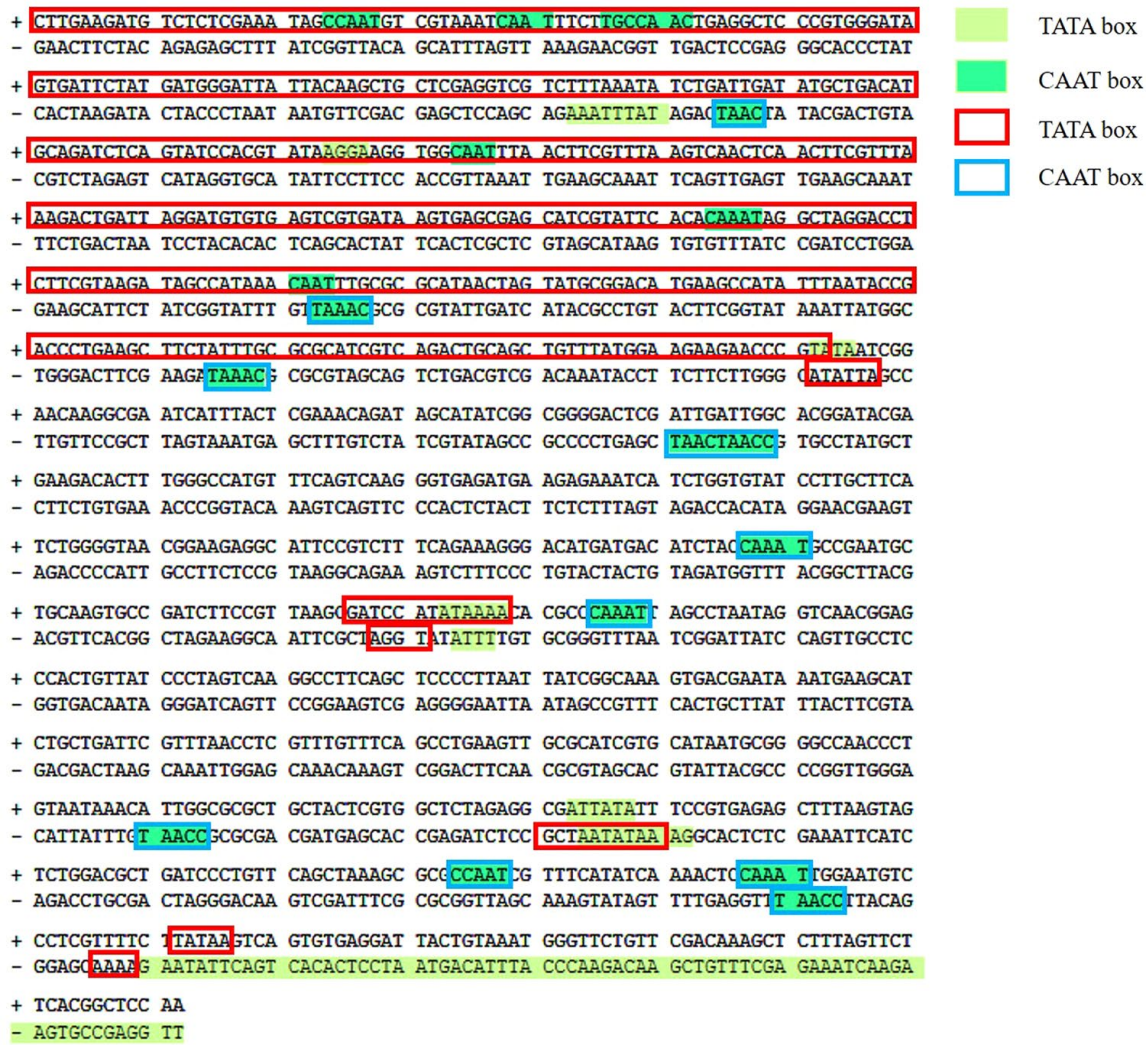
*In silico* identification of core and cis-regulatory elements in Pxy sequence. The 1063 bp Pxy promoter (in contig1365 with GenBank accession no AMGP01001365) sequence analysis depicts presence of putative *cis*-acting elements in both orientations determined by PLACE^45^ (https://www.dna.affrc.go.jp/PLACE/?action=newplace) and PlantCARE^46^ (http://bioinformatics.psb.ugent.be/webtools/plantcare/html/) online software.

The 1063 bp of intergenic region (Pxy promoter) was amplified from the *Foc* genomic DNA of using primers (PxyFw and PxyRv; Table 1) and digested using *Not*I and *Nhe*I sites. This amplicon was cloned along with EGFP gene and the glucoamylase terminator region into pCSN44 vector backbone using three-way ligation followed by sequencing of the fragment. The promoter sequence of Pxy was used as a query to search the plant *cis*-acting regulatory elements database for identifying the core and *cis*-regulatory elements. Stretches of TATA box elements were present on both plus and minus strand of the Pxy promoter sequence (Fig. 1). Several CAAT boxes were also identified in the Pxy promoter sequence at discrete locations (Fig. 1). *cis*-Regulatory elements such as ABRE, Myb and W-box were also present in the Pxy promoter sequence. The Pxy sequence was used as a query in the *Foc* whole genome shotgun contigs database nucleotide blast program (NCBI) to identify its closest homolog. Among the six hits obtained, the race 1 isolate, *Fusarium oxysporum* f. sp. *cubense* strain 160527 gave 100% query cover with a 99.91% identity^29^. Ninety five percent identity was seen when Pxy sequence was aligned with race 1 (*Fusarium oxysporum* f. sp. *cubense* race 1 contig1365,) and race 4 (*Fusarium oxysporum* f. sp. *cubense* race 4 contig828) homologs. The multiple sequence alignment of these three sequences was carried out using MultAlin (http://multalin.toulouse.inra.fr/multalin/) online software (Supplementary Fig. S1). Further when an open blast was performed, Pxy homologs were identified in different *formae speciales* of *Fusarium oxysporum* with 100% query cover and up to 95% identity. These FASTA sequences were retrieved and used for multiple sequence alignment using MultAlin online software (Supplementary Fig. S2).

### Genetic modification of *Foc*

**Figure 2:**
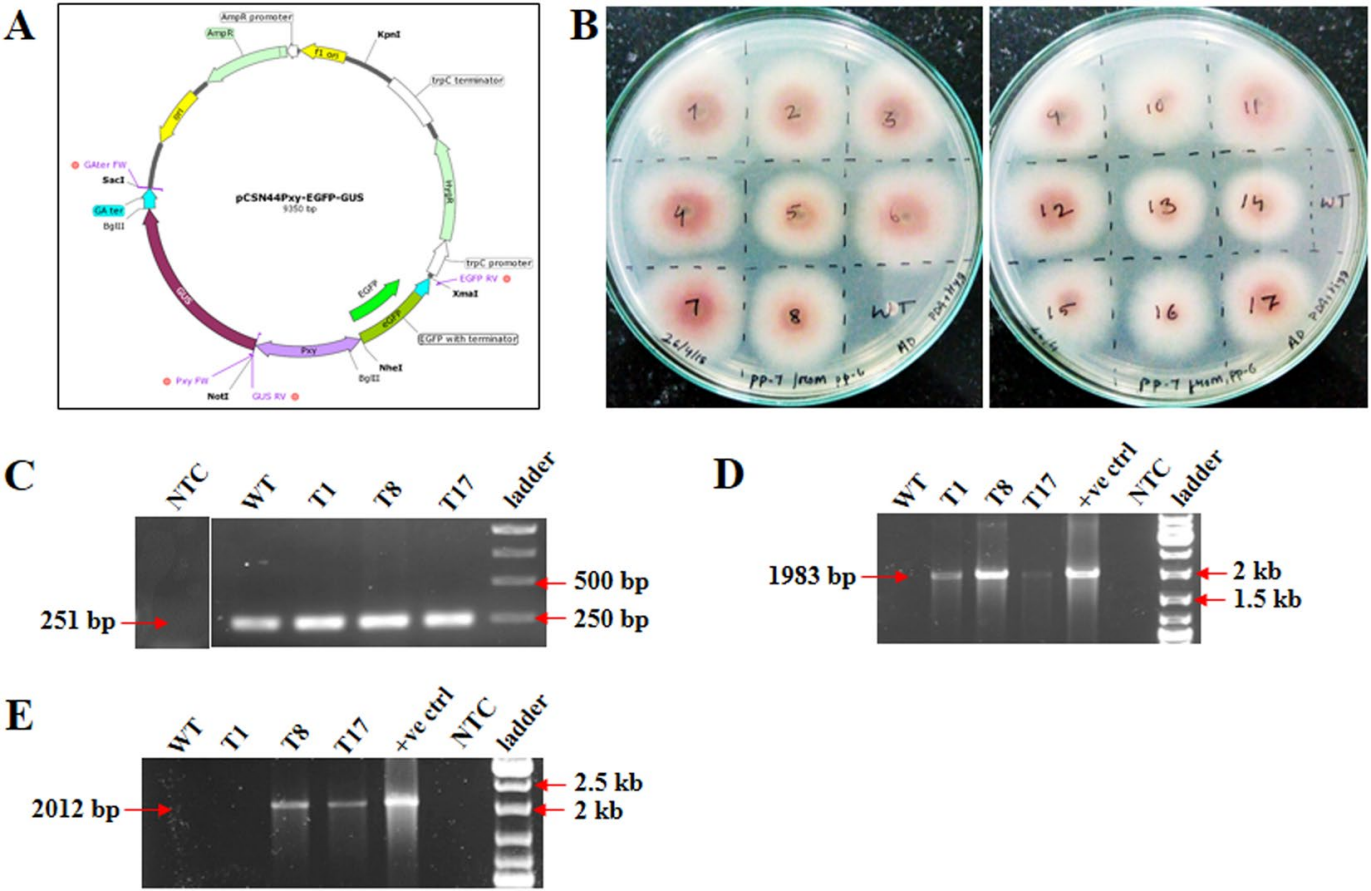
Genetic modification of *Fusarium oxysporum* f. sp. *cubense* (*Foc*). (**A**) The vector map generated using SNAPGENE tool showing the Pxy promoter sequence cloned between β-glucuronidase and EGFP cassette. The primer set (marked as red solid circle) used for screening the transformants are depicted in the vector map. (**B**) *Foc* transformants growing on hygromycin containing medium. (**C**) PCR amplification of 251 bp *Foc* TEF1α gene fragment to determine the quality of the extracted genomic DNA for further PCR analysis. A single band of 251 bp was observed in WT, T1, T8 and T17 genomic DNA samples. (**D**,**E**) Genomic DNA PCR of the transformants T1, T8 and T17 to determine the integration of the plasmid DNA into the *Foc* genome using primers (Table 1) to amplify 1983 bp and 2012 bp sequence. There was no amplification observed in the WT genomic DNA sample. [NTC-non template control, +ve ctrl-positive control, ladder-1 Kb gene ruler].

Seventeen transformants were obtained in hygromycin supplemented medium and these were phenotypically comparable with the WT strain (Fig. 2B). These transformants were sub-cultured for seven generations in the medium containing hygromycin. Of these, three transformants (T1, T8 and T17) were screened by genomic DNA PCR. To check for the quality of the genomic DNA extracted from WT and all the transformants, PCR was performed using TEF1α primers (Table 1) to amplify 251 bp region (Fig. 2C). Further, the amplicons of 1983 bp corresponding to Pxy promoter plus EGFP coding sequence along with glucoamylase gene terminator and 2012 bp corresponding to β-glucuronidase coding sequence and glucoamylase gene terminator was amplified from T8 and T17 transformants (Fig. 2D,E). However, the T1 transformant showed no amplification of the 2012 bp fragment and was therefore not used for further analysis. Both these PCR amplicons were absent in the WT strain. For all further experiments the T17 transformant was used.

### β-glucuronidase analysis

**Figure 3:**
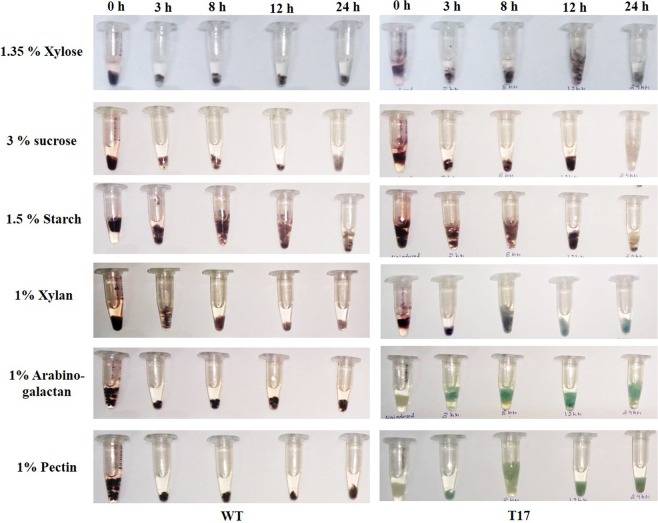
Histochemical β-glucuronidase (GUS) staining of the *Fusarium oxysporum* f. sp. *cubense* (*Foc*) at different time points. Expression of β-glucuronidase gene at different time point was determined using histochemical β-glucuronidase staining of *Foc* grown in medium containing different carbon sources. Indigo coloration was seen in *Foc* T17 strain grown in medium containing xylan, arabinogalactan and pectin, whereas no change was seen in WT strain grown under all conditions and in T17 strain grown in xylose, sucrose and starch containing medium.

**Figure 4:**
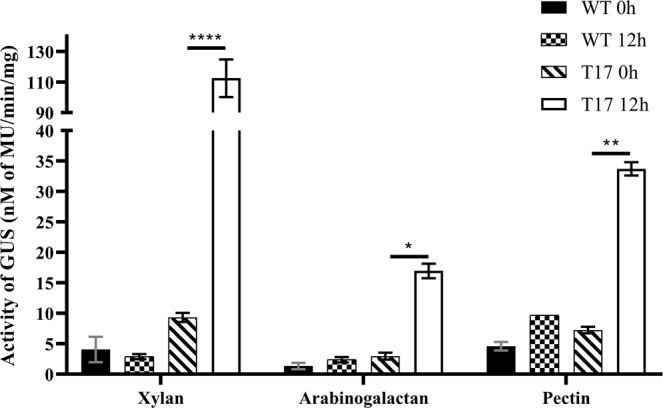
Fluorimetric determination of β-glucuronidase (GUS) activity in *Fusarium oxysporum* f. sp. *cubense* (*Foc*) using 4-Methyl Umbelliferyl β-D-Glucuronide (MUG) assay. Quantitative analysis of GUS expression in *Foc* strains at 12 h time point in medium containing xylan, arabinogalactan and pectin using fluorimetric MUG assay. Two-way ANNOVA Tukey’s multiple comparison test was performed using GraphPad Prism 8.0.2. (*P < 0.05; **P < 0.01; ****P < 0.0001).

In the histochemical staining method, the enzyme GUS reacts with 5-Bromo-4-chloro-3-indolyl-β-D-Glucuronide (X-Gluc) substrate to 5-Bromo-3-chloro-indole which upon oxidative dimerization becomes indigo. The WT and T17 transformant grown on different carbon source containing media for 3, 8, 12 and 24 h were individually incubated in GUS buffer overnight at 37 °C. The WT strain grown in different carbon sources containing medium did not show any indigo coloration, indicating that there is no GUS activity. Additionally, no GUS activity was observed in the T17 protein lysate samples collected at zero-hour time point. The mycelia of T17 grown in xylan, arabinogalactan and pectin showed indigo coloration at all four time points tested, demonstrating the presence of GUS enzyme (Fig. 3). This indicated that the promoter was inducible with xylan, arabinogalactan and pectin thereby transcribing the GUS gene resulting in the functional enzyme. It may be noted that, there was no β-glucuronidase activity observed in the T17 mycelia grown in the medium supplemented with xylose, sucrose and starch. Further, to assess the amount of β-glucuronidase activity, MUG assays at 0 and 12 hours for all the samples was performed. In the MUG assays, 4-Methyl-umbelliferyl- β-D-glucuronide is converted to β-glucuronide and 4-Methyl-umbelliferone (4-MU) by the action of β-glucuronidase enzyme. The 4-Methyl-umbelliferone is a fluorochrome with an excitation and emission at 365 nm and 465 nm, respectively. The fluorescence intensity was measured by individually mixing the protein lysates from WT and T17 mycelia with MUG substrate for varying time periods (10 to 70 min). The β-glucuronidase specific activity was calculated in terms of the amount of 4-MU produced per milligrams of total protein per minute. There was no significant β-glucuronidase activity observed in WT grown in medium containing xylan, arabinogalactan and pectin in protein lysates from both 0 and 12 h time points. Activity was also not observed in protein lysates from the T17 transformant at 0 h time point. However, β-glucuronidase activity was significantly induced in the T17 transformant grown in medium supplemented with xylan followed by lesser activity in pectin and least in arabinogalactan (Fig. 4). This indicated that the β-glucuronidase activity is triggered by xylan, arabinogalactan and pectin.

### EGFP analysis

**Figure 5:**
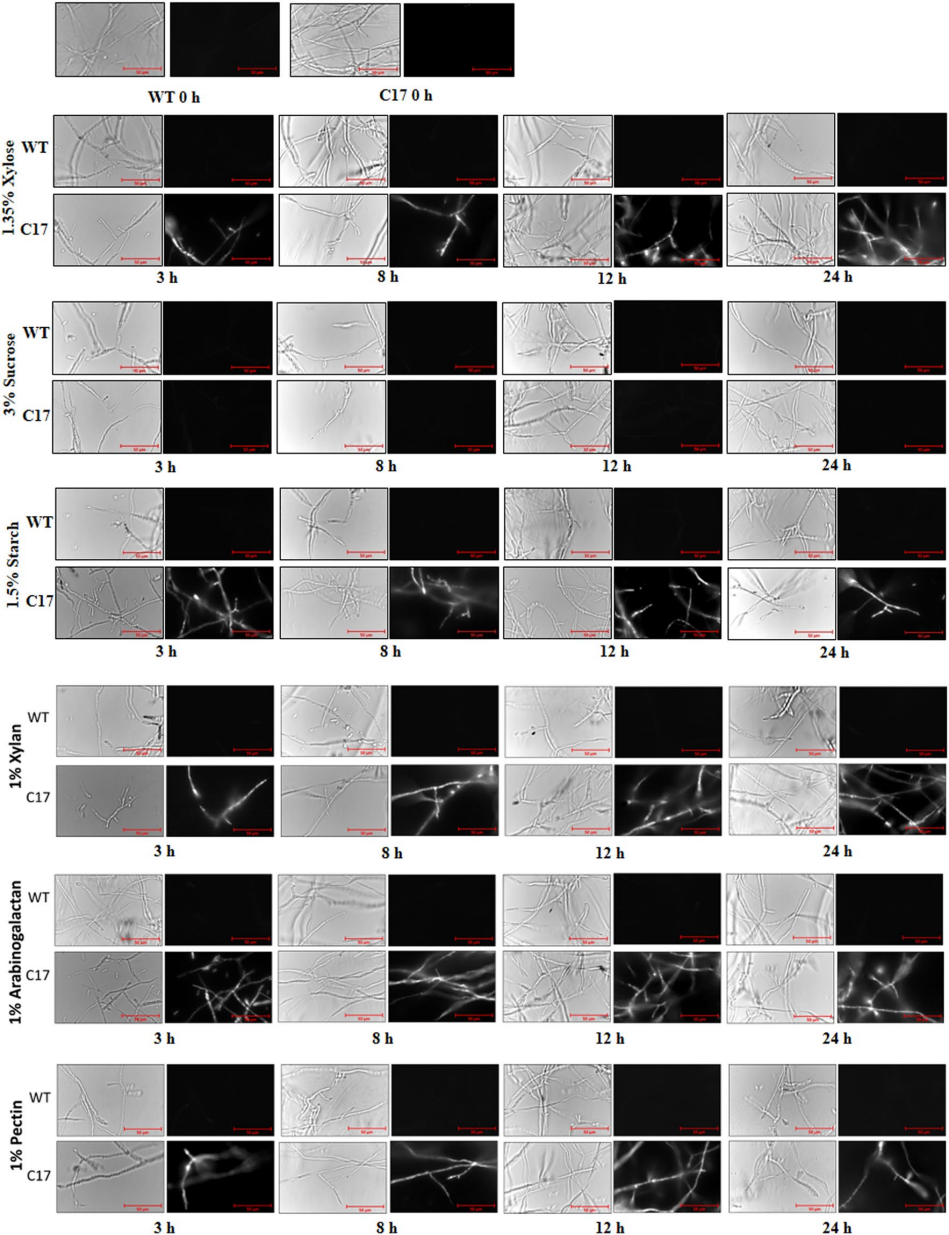
Enhanced green fluorescent protein (EGFP) visualization using fluorescence microscopy. Expression of EGFP gene at different time point was determined using fluorescence microscopy of *Fusarium oxysporum* f. sp. *cubense* (*Foc*) grown in medium containing different carbon sources. Fluorescence was seen in *Foc* T17 strain grown in medium containing xylan, arabinogalactan, starch, xylose and pectin, whereas no change was seen in WT strain grown under all conditions and in T17 strain grown in sucrose containing medium. Bar represents 50 µm.

**Figure 6:**
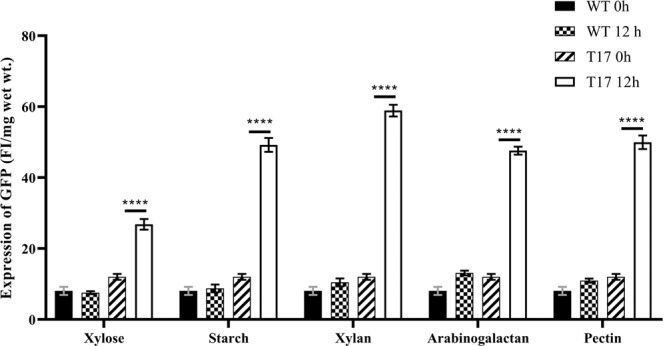
Fluorescence intensity determination of Enhanced green fluorescent protein (EGFP) expressed in *Fusarium oxysporum* f. sp. *cubense* (*Foc*). Quantitative analysis of EGFP expression in *Foc* strains at 12 h time point in minimal medium containing xylan, arabinogalactan, starch, xylose and pectin as sole carbon source using fluorimeter. Two-way ANNOVA Tukey’s multiple comparison test was performed using GraphPad Prism 8.0.2. (****P < 0.0001).

The *Foc* WT and T17mycelia grown in minimal medium supplemented with xylan, arabinogalactan, pectin, starch, sucrose, xylose as carbon source for different time points (0, 3, 8, 12 and 24 h) was observed under fluorescence microscope using FITC filter having excitation at 488 nm and emission at 509 nm. The EGFP expression was seen in the mycelia grown in medium containing xylan, arabinogalactan, pectin, starch, xylose as carbon source; but, not in sucrose containing medium (Fig. 5). This clearly indicates that the Pxy promoter is triggered by all these carbon sources except sucrose. The EGFP fluorescence that corresponds to the amount of EGFP expression was quantitated using fluorimeter. After measuring the fluorescence intensity, the mycelial wet weight was determined and expressed as FI/mg of mycelia. The T17 transformant showed maximum fluorescence in xylan-containing medium followed by starch, arabinogalactan, pectin and xylose in decreasing order (Fig. 6). This indicates that the Pxy promoter is regulated by carbon sources that are an important component of the plant cell wall.

## Discussion

Plant pathogenesis is a complex series of events that are triggered when a pathogen encounters its host plant. During this interaction, the pathogen secretes an array of proteins, enzymes and toxins that prevent host defense response and favors pathogen survival and multiplication culminating into a disease^30,31^. The protein coding genes are triggered by a signaling cascade which perceive plant molecules as signals to initiate pathogenesis. Thus, it is likely that all those genes required during pathogenesis are regulated at the promoter level by a few signaling molecules^32^. The regulation of the pathogenicity genes can be economized if there is a single regulatory sequence that controls the expression^18,33^. However, such a phenomenon is quite common in prokaryotes where the operon system is established^34–36^. Nonetheless, in eukaryotes, at least two genes can be controlled by a single regulatory sequence (called as intergenic region) that controls the expression of two flanking genes.

In the present study, we identified such an intergenic region of 1063 bp that lies between two genes coding for putative beta-xylosidase (accession number: ENH64116) and arabinoxylan arabinofuranohydrolase (accession number: ENH64117). These genes belong to the glycosyl hydrolase family 43 and are known to play an important role in the breakdown of xylan which is an important component of plant cell wall. Moreover, xylanases play a significant role in both penetration and horizontal expansion of phytopathogenic fungi in infected plants^37,38^. Thus, a solitary signaling molecule from the host plant can simultaneously trigger the expression of these two genes, involved in xylan metabolism.

Bidirectional promoters are well studied in prokaryotes^3^, humans^4,39,40^, plants^41^, yeasts^5,11^ and insects^7^. However, there are only two reports on identification of bidirectional promoters from filamentous fungi. The *niaD* and *niiA* genes of *Aspergillus nidulans* coding for nitrate and nitrite reductases are divergently transcribed and are separated by a 1200 bp intergenic region. These genes are under the control of the positively acting *NirA* transcription factor, which mediates nitrate utilization^42^. Three penicillin biosynthesis genes *pcbAB*, *pcbC*, and *penDE* are arranged as a cluster in *Penicillium chrysogenum*, *Aspergillus nidulans* and *Penicillium nalgiovense*. Among these three genes, the expression of *pcbAB* and *pcbC* genes are controlled by a 1.16 kb bidirectional promoter region regulated by nutritional and developmental factors^19,43^. This indicates that the genes involved in a common metabolic pathway are clustered together and a maximum of two genes are regulated by an intergenic region. The Pxy promoter isolated in this study is comparable to the two promoters isolated from *Aspergillus* and *Penicillium*. Further, the Pxy promoter controls the expression of genes required for xylan metabolism and is an important element in pathogenesis.

For characterization, the Pxy promoter sequence was cloned in pCSN44 vector in between two reporter genes (β-glucuronidase and EGFP) and the bidirectionality of the intergenic sequence was tested by assaying the gene products. This strategy was also adopted in plants such as rice and *Arabidopsis*, wherein two reporter genes (GUS and GFP) were cloned across the intergenic region^15,41^. In the current study, Pxy promoter sequence was inducible as there was no expression noticed in the zero hour time point irrespective of the media in which the transformant is grown. The reporter signals were observed after 3 h of incubation in the medium containing different carbon sources. Since this promoter controls the expression of gene products involved in xylan metabolism, we tested xylan and xylose as the most probable inducers. However, β-glucuronidase and EGFP expression was highest in the xylan-containing medium, but only EGFP signals were observed in xylose medium. This indicated that the Pxy promoter sequence differentially regulated the reporter gene pair. Moreover, other plant sugars such as starch, arabinogalactan, pectin and sucrose were further tested as inducers for Pxy sequence. β-glucuronidase activity was maximum in pectin followed by arabinogalactan, whereas no activity was seen in starch and sucrose. The EGFP fluorescence was observed in pectin, starch and arabinogalactan in a decreasing order of intensity. Since, EGFP fluorescence was differently expressed with starch and xylose than that of the β-glucuronidase activity, the Pxy sequence appeared to differentially regulate the reporter gene pair. The *in planta* Pxy promoter activity was detected by infecting the susceptible cultivars of banana (cv. Rasthali) with WT and T17 strains. The RNA was extracted from the infected roots after 18 h, converted into cDNA and used as template for quantitative real-time PCR. The β-glucuronidase and EGFP expression (using TEF1α as normalization gene control) was 9- and 7-fold, respectively in the T17 transformant as compared to the WT strain *in planta* (Supplementary Fig. S3). These results suggested that Pxy promoter sequence is activated during infection. Bidirectional promoters are known to be regulated in specific tissues at particular developmental stage or triggered during stress of hormonal stimulation^8,44^.

In synthetic biology and genetic engineering, the strength of the promoter is crucial to drive the expression of the heterologous protein. The promoter strength can be fairly determined by studying the binding affinity of the transcription factors to its sequence or assessing the expression level of the gene driven by the promoter. In our study, we determined the expression levels using quantitative real-time PCR, and found that the promoter Pxy is directionally biased. The relative expression of the EGFP and GUS genes showed no difference when xylan was used as an inducer (data not shown). Such bidirectional regulatory sequences find applications in genetic and metabolic engineering where the need for cumulative co-expression and stacking of two genes arises.

Wiemann and co-workers^21^ used an inducible *niiA*/*niaD* promoter sequence to drive the expression of sterigmatocystin Zn(II)2Cys6 transcription factor-encoding gene *aflR* and its cofactor *aflS*. These two factors regulate the expression of *Aspergillus nidulans* endogenous secondary metabolites genes to produce sterigmatocystin. Thus, a single promoter was used to regulate 8 genes from the sterigmatocystin gene cluster. Therefore, if the regulatory sequence is an inducible one it’s highly valuable for expressing heterologous proteins. The Pxy promoter sequence isolated in this study is a compact and inducible promoter which is regulated by plant sugars and can be readily used for expressing heterologous proteins in filamentous fungi.

## Supplementary information

Supplementary Figures S1-S3.
