# Supplementary material for: Functional characterization of an inducible bidirectional promoter from Fusarium oxysporum f. sp. cubense
Source: Sci Rep. 2020 Feb 11;10:2323. doi: 10.1038/s41598-020-59159-0 (PMC7012866; doi:10.1038/s41598-020-59159-0)
Supplement: Supplementary file 1 — Supplementary Figures S1-S3. [file 41598_2020_59159_MOESM1_ESM.pdf]

Functional characterization of an inducible bidirectional promoter from *Fusarium oxysporum* f. sp. *cubense*

Ashutosh Dash, Vartika Gurdaswani, Jacinta S. D'Souza and Siddhesh B. Ghag*
School of Biological Sciences, UM-DAE Centre for Excellence in Basic Sciences,
Kalina campus, Santacruz (East), Mumbai - 400098, INDIA
*Corresponding author: *siddhesh.ghag@cbs.ac.in*

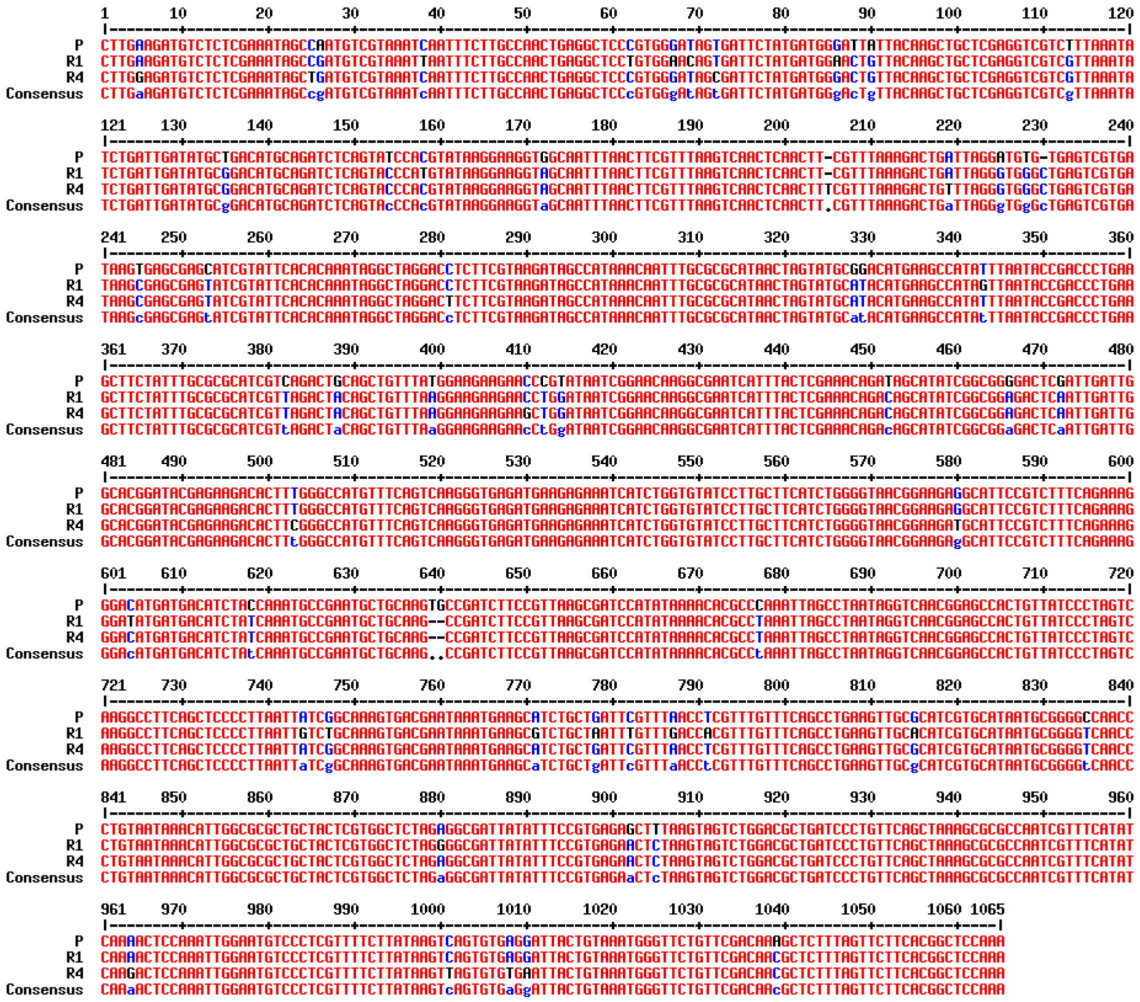

Supplementary Fig S1: Multiple sequence alignment of Pxy sequence with race 1 (R1) strain (*Fusarium oxysporum* f. sp. cubense race 1 contig1365) and race 4 (R4) strain (*Fusarium oxysporum* f. sp. *cubense* race 4 contig828) homologs using multalign online software.

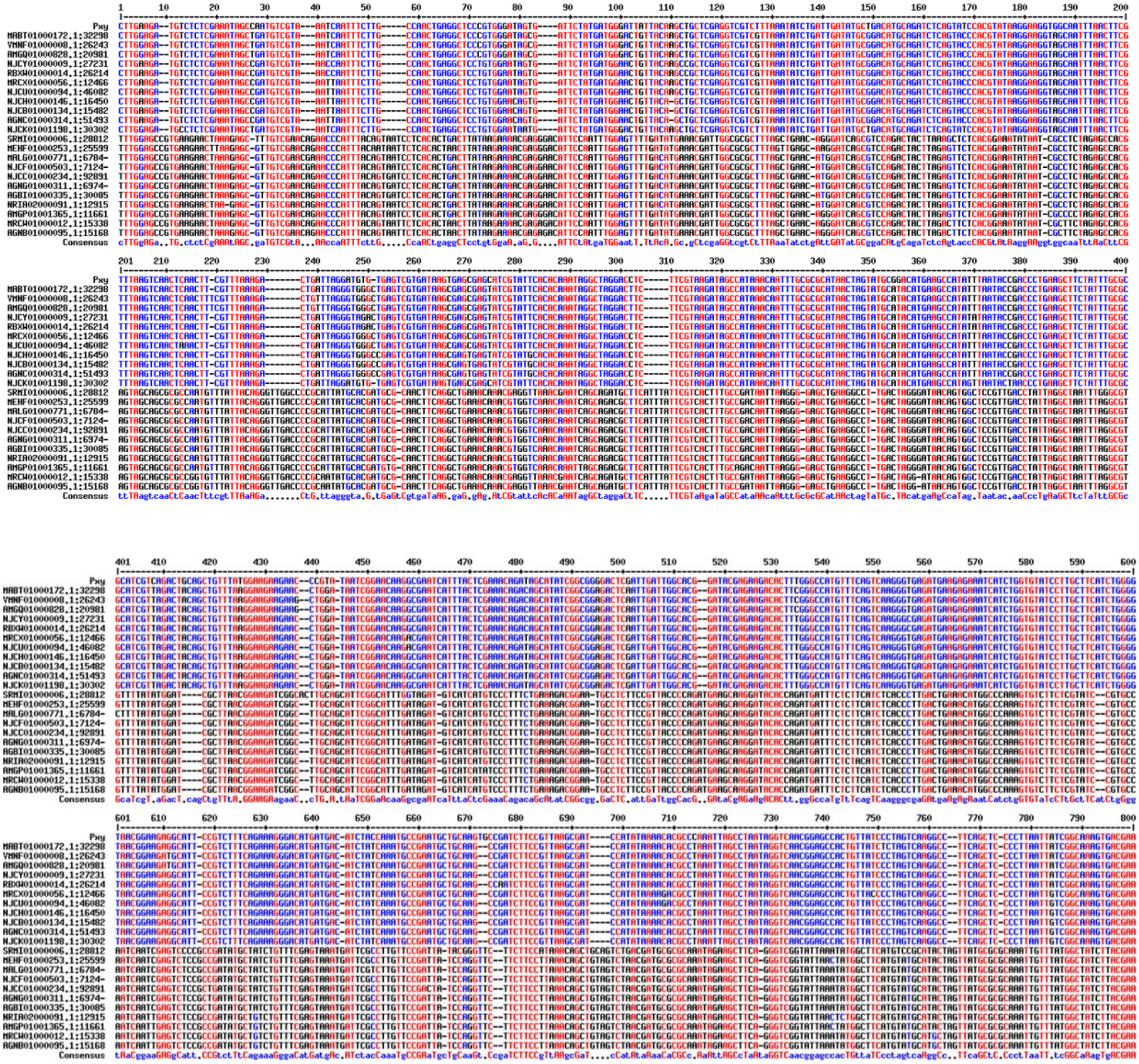

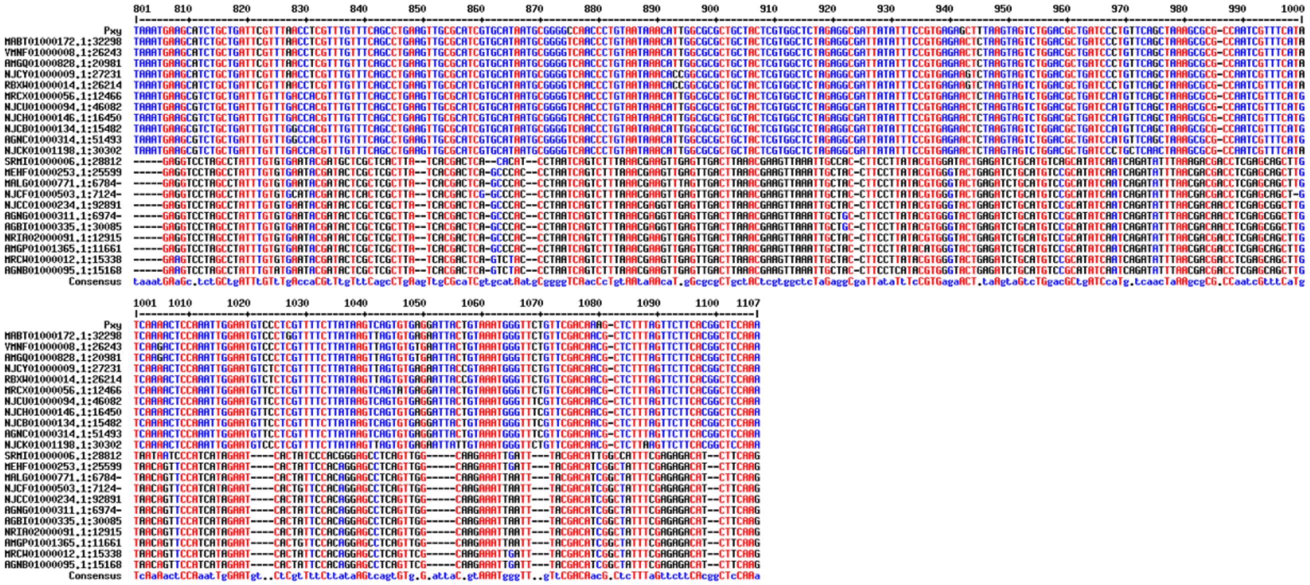

Supplementary Fig. S2: Multiple sequence alignment of Pxy homologous sequence from different formae speciales of *Fusarium oxysporum*. The sequences used for alignment were SRMI01000006.1:288126-289188 *Fusarium oxysporum* f. sp. *cubense* strain 160527 Focb_160527_v1_contig10, MABT01000172.1:322987-324048 *Fusarium oxysporum* f. sp. *cucumerinum* strain Foc011 Foq011_contig_172, NJCK01001198.1:30302-31362 *Fusarium oxysporum* f. sp. *gladioli* strain G76 Fogla_G76_contig_1198, VMNF01000008.1:2624398-2625460 *Fusarium oxysporum* f. sp. *cubense* strain TR4 isolate UK0001 scf_28419_8, AMGQ01000828.1:20981-22043 *Fusarium oxysporum* f. sp. *cubense* race 4 contig828, MRCX01000056.1:124667-125728 *Fusarium oxysporum* strain Fo_A13 contig_56, MEHF01000253.1:25599-26660 *Fusarium oxysporum* f. sp. *ciceris* strain 38-1 Scaffold_0253, NJCU01000094.1:460820-461881 *Fusarium oxysporum* f. sp. *nicotianae* strain Ft-1512 Fonic_012_contig_94, MALG01000771.1:6784-7845 *Fusarium oxysporum* f. sp. *niveum* strain Fon021 Fon021blob3c_contig_771, NJCY01000009.1:2723115-2724176 *Fusarium oxysporum* f. sp. *melonis* 26406 Fom001_contig_9, NJCC01000234.1:92891-93952 *Fusarium oxysporum* f. sp. *melongenae* strain J-71 Fomel_001_contig_234, MRCW01000012.1:15338-16398 *Fusarium oxysporum* f. sp. *cepae* strain FoC_A23 contig_12, AGNG01000311.1:6974-8035 *Fusarium oxysporum* f. sp. *raphani* 54005 cont1.311, AGBI01000335.1:30085-31146 *Fusarium oxysporum* f. sp. *pisi* HDV247 cont1.335, RBXW01000014.1:2621432-2622493 *Fusarium oxysporum* f. sp. *lycopersici* strain race 3 isolate D11 chromosome 11, NRIA02000091.1:129151-130211 *Fusarium*

oxysporum f. sp. *conglutinans* strain FGL03-6 Scaffold20_2, NJCH01000146.1:164503-165563 *Fusarium oxysporum* f. sp. *lagenariae* strain Lag:3-1 Folag_004_contig_146, NJCF01000503.1:7124-8184 *Fusarium oxysporum* f. sp. *lilii* strain Fol39 Folil_Fol39_contig_503, AMGP01001365.1:11661-12722 *Fusarium oxysporum* f. sp. *cubense* race 1 contig1365, NJCB01000134.1:154828-155888 *Fusarium oxysporum* f. sp. *momordicae* strain NRRL26413 Fomom_001_contig_134, AGNC01000314.1:51493-52553 *Fusarium oxysporum* f. sp. *vasinfectum* 25433 cont1.314, AGNB01000095.1:151683-152743 *Fusarium oxysporum* f. sp. *radicis-lycopersici* 26381 cont1.95.

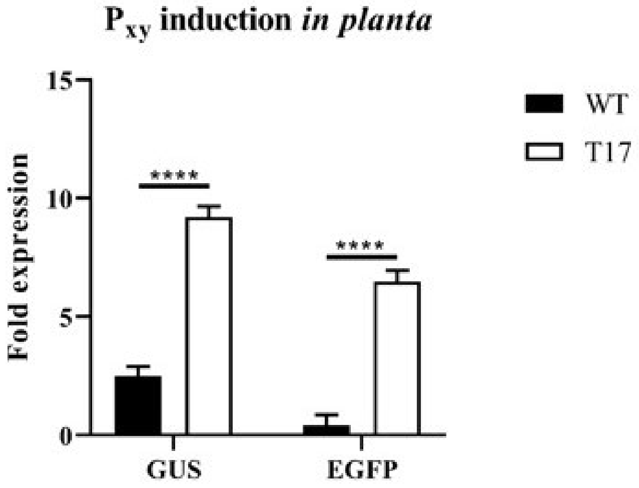

Supplementary Fig. S3: Detection of *in planta* activation of Pxy promoter sequence using quantitative real-time PCR. Susceptible banana plants were inoculated with spore suspension (10^7 spores/ mL) of WT and T17 strain individually. The infected roots were collected and total RNA was extracted using RNeasy Plant Mini Kit (Qiagen, Germany). The total RNA was converted into cDNA using Oligo-dT primers and ProtoScript® First Strand cDNA Synthesis Kit (NEB, MA, USA). Quantitative real-time PCR was performed using gene specific primers for amplification of β-glucuronidase and EGFP gene along with the translational elongation factor 1α gene as normalization control. A ~9-fold and ~7-fold induction was seen in β-glucuronidase and EGFP, respectively, in the T17 strain as compared to the wild-type strain *in planta*.
